# Supplementary material for: Detecting and quantifying networks of biological kinship via exponential family random graph models
Source: Genetics. 2026 Feb 26;232(4):iyag053. doi: 10.1093/genetics/iyag053 (PMC13050185; doi:10.1093/genetics/iyag053)
Supplement: iyag053_Supplementary_Data [file iyag053_supplementary_data.pdf]

Detecting and Quantifying Networks of Biological Kinship via Exponential Family Random Graph Models

Adam B. Rohrlach[1,2,*,†], Guido Alberto Gnecchi-Ruscone[3,4], Zuzana Hofmanová[1,5], Zsófia Rácz[6], Matthew Roughan[7], Wolfgang Haak[1] and Jonathan Tuke[7,†]

[1]Department of Archaeogenetics, Max Planck Institute for Evolutionary Anthropology, Deutscher Platz 6 04103, Leipzig, Germany
[2]School of Biological Sciences, University of Adelaide, Adelaide, North Terrace campus 5005, Australia
[3]Archaeo- and Palaeogenetics, Institute for Archaeological Sciences, Department of Geosciences, University of Tübingen, Geschwister-Scholl-Platz 72074, Tübingen, Germany
[4]Senckenberg Centre for Human Evolution and Palaeoenvironment at the University of Tübingen, Geschwister-Scholl-Platz 72074, Tübingen, Germany
[5]Department of Archaeology and Museology, Masaryk University, Žerotínovo nám. 617/9, 601 77 Brno, Czechia
[6]Institute of Archaeological Sciences, ELTE - Eötvös Loránd University, Múzeum krt. 4/B, 1088 Budapest, Hungary
[7]School of Mathematical and Computer Sciences, University of Adelaide, North Terrace campus 5005, Adelaide, Australia
[†]These authors contributed equally to this work.

[*]Corresponding author: Department of Archaeogenetics, Max Planck Institute for Evolutionary Anthropology, Deutscher Platz 6 04103, Leipzig, Germany.
Email: adam_ben_rohrlach@eva.mpg.de

S1. Interpretation of coefficients

We give these examples below in which models *only* use site as a predictor for genetic relatedness, where x_i is the variable which indicates the site at which individual i was buried.

1. **Null Model**: site location does not affect the probabilities of two individuals being connected, and hence connectedness is simply random.

$$\log\left(\frac{p_{ij}}{1-p_{ij}}\right) = \theta_0.$$

Note then that θ_0 is the log-odds of two random individuals sharing a genetic connection.

2. **Homophily**: All that matters is whether the individuals are from the same site, i.e

$$\log\left(\frac{p_{ij}}{1-p_{ij}}\right) = \theta_0 + \theta_1 z_{ij},$$

where

$$z_{ij} = I\left(x_i = x_j\right).$$

Here, θ_0 is the log-odds that two individuals from *different* sites share a genetic connection, and θ_1 is the amount that the log-odds increase (or decrease) when individuals are buried at the same site.

3. **Differential Homophily**: Whether two individuals are buried at the same site, and which site this is, is of importance, i.e.

$$\log\left(\frac{p_{ij}}{1-p_{ij}}\right) = \theta_0 + \sum_{k=1}^{3}\theta_k z_{ijk}$$

where

$$\begin{aligned} z_{ij1} &= I\left(x_i = A \cap x_j = A\right), \\ z_{ij2} &= I\left(x_i = B \cap x_j = B\right), \\ z_{ij3} &= I\left(x_i = C \cap x_j = C\right). \end{aligned}$$

Hence, again θ_0 is the log-odds that two individuals from *different* sites share a genetic connection, and θ_1, θ_2 and θ_3 represent the amount the log-odds increase (or decrease) when individuals are both buried at site A, B or C, respectively.

4. **Attribute Mixing**: the combination of the sites that the two individuals are buried at is of importance.

$$\log\left(\frac{p_{ij}}{1-p_{ij}}\right) = \theta_0 + \sum_{k=1}^{5}\theta_k z_{ijk},$$

where

$$\begin{aligned} z_{ij1} &= I\left(x_i = A \cap x_j = A\right), \\ z_{ij2} &= I\left(x_i = B \cap x_j = B\right), \\ z_{ij3} &= I\left(x_i = C \cap x_j = C\right), \\ z_{ij4} &= I\left(x_i = A \cap x_j = C\right) + I\left(x_i = C \cap x_j = A\right), \\ z_{ij5} &= I\left(x_i = B \cap x_j = C\right) + I\left(x_i = C \cap x_j = B\right). \end{aligned}$$

Hence, θ_0 is the log-odds of two random individuals buried at sites A and B, and the θ_k give the increase (or decrease) in the log-odds if the individuals are both buried at site A, B or C, or at sites A and C, or sites B and C, respectively.

S2. Simulated model descriptions

To test the ability of ERGMs to differentiate between different and known model types, we simulated 100 independent realisations of each of the following models. Overall, we allowed nodes to have one of two attributes. First, genetic sex can take one of two values: “Sex 1” or “Sex 2”. Second, archaeological site (referred to as "site") can take one of three values: A, B or C.

A. The null model: $\mathcal{M}_0$

The null model is a model in which no variable explains the connections between nodes (Figure S1). The genetic sex and the site are not predictors of when two individuals are connected.

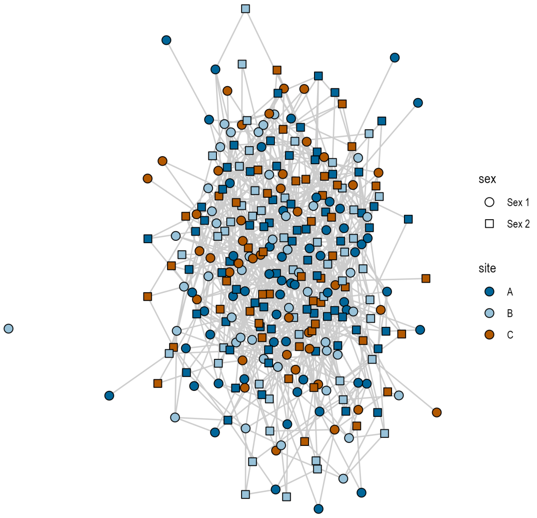

Figure S1 A single realisation of the null model, $\mathcal{M}_0$.

Here we set $\theta_0 = -4$, and all other values of $\theta_i = 0$. This corresponds to a probability of approximately 0.0183 of any two random individuals being related. This is the base probability used in all models.

B. The site match model: $\mathcal{M}_1$

The site match model makes any two individuals equally more likely to share a connection, no matter which site they are attributed to Figure S2. This causes the individuals to cluster by site, and those clusters look equally densely connected.

Here, $\theta_1 = \theta_2 = \theta_3 = 3$, corresponding to 14.95-fold increase in the probability of individuals being connected if they share the same burial site.

C. The differential site match model: $\mathcal{M}_2$

The differential site match model makes two individuals more likely to share a connection if they are buried at the same site, but not equally, and the increase depends on *which* site they are both buried at (Figure S3). This causes the individuals to cluster by site, and for those clusters to look more densely connected depending on the scale of the increase.

Here, $\theta_1 = \theta_2 = 3$, corresponding to 14.95-fold increase in the probability of individuals being connected if they share the same burial site, and are buried at site A or B. However

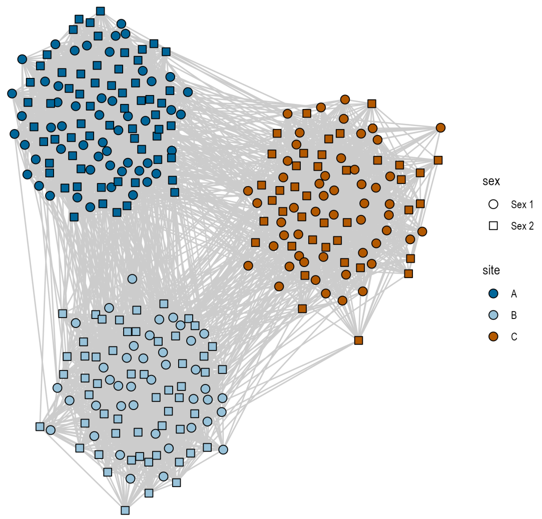

Figure S2 A single realisation of the site match model, $\mathcal{M}_1$.

$\theta_3 = 4$, meaning that if both individuals are buried at C, then the probability is increased to 27.79-fold, compared to individuals buried at different sites.

D. The site mix model: $\mathcal{M}_3$

The site mix model makes two individuals equally more likely to share a connection if they are buried at the same site, similar to model $\mathcal{M}_1$ (Figure S4). However, it also allows sites A and C to be more connected than sites, with no additional probability for connections between sites A and B, or sites B and C.

Here, $\theta_1 = \theta_2 = \theta_3 = 3$, corresponding to 14.95-fold increase in the probability of individuals being connected if they share the same burial site. However, there is still a 6.62-fold increase in the probability of a connection between individuals at sites A and C, resulting in a network in which sites A and C appear to “overlap”, which site B is more pronounced in its separation.

E. The sex match model: $\mathcal{M}_4$

The sex match model makes two individuals equally more likely to share a connection if they are of the same genetic sex (Figure S5). This is similar to the site match model, and is included to test models where the variables has only two possible levels.

Here, $\theta_6 = \theta_7 = 0.75$, corresponding to 2.07-fold increase in the probability of individuals being connected if individuals share the same genetic sex. This is an unlikely scenario and is included for comparison to the three-level site match model.

F. The differential sex model: $\mathcal{M}_5$

The differential sex model makes two individuals more likely to share a connection if they are of the same genetic sex, but this differs for different levels (Figure S6). If one of the coefficients were negative, this could be a result of matrilocality or patrilocality.

In this case, if two individuals are both Sex 2, then they have they have a 4.21-fold increase in the probability of individuals being connected, but only a 2.07-fold increase if both are Sex 1.

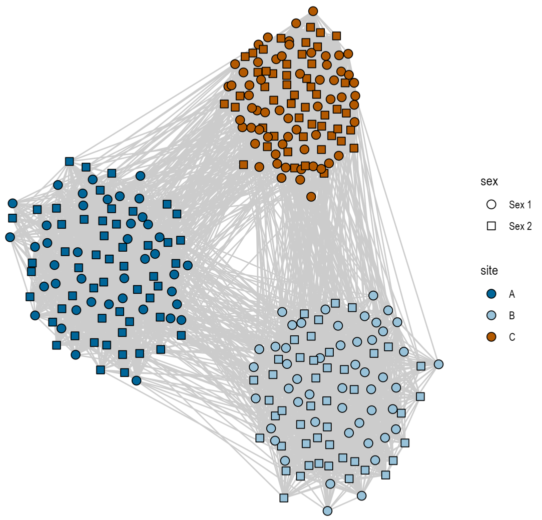

Figure S3 A single realisation of the differential site match model, $\mathcal{M}_2$.

Model ID	θ_0	θ_1	θ_2	θ_3	θ_4	θ_5	θ_6	θ_7
$\mathcal{M}_0$	-4	0	0	0	0	0	0	0
$\mathcal{M}_1$	-4	3	3	3	0	0	0	0
$\mathcal{M}_2$	-4	3	3	4	0	0	0	0
$\mathcal{M}_3$	-4	3	3	3	2	0	0	0
$\mathcal{M}_4$	-4	0	0	0	0	0	0.75	0.75
$\mathcal{M}_5$	-4	0	0	0	0	0	0.75	1.5
$\mathcal{M}_6$	-4	3	3	3	0	0	0.75	0.75

Table S1 Coefficient values of θ_i which differentiate the different models, $\mathcal{M}_0, \cdots, \mathcal{M}_6$.

G. The mixed homophily model: $\mathcal{M}_6$

The mixed homophily makes both site location and genetic sex contribute to the probability of two individuals sharing a connection (Figure S7). The effect of sharing a site is greater than the effect of having the same genetic sex.

This model is the most complicated, but allows for the analysis of the effect of genetic sex, while still *accounting* for the effect of site. This might be of importance when there is a sampling bias at one site, say when yielding more individuals of Sex 1 at site A and more of Sex 2 at site B.

S3. Simulated model parameters

A concise table of model parameters are given below for the model equation

$$\log\left(\frac{p_{ij}}{1-p_{ij}}\right) = \theta_0 + \sum_{k=1}^{6} \theta_k z_{ijk},$$

differentiating the models from Supplementary Section S2.

Note that we calculate the fold-change in probability from the coefficient values in the following way. For an coefficient value of θ', and a baseline probability θ_0 (the coefficient associated with "edges") the fold-change, ϕ' can be found via

$$\phi' = \frac{e^{\theta_0+\theta'} \big/ \left(1+e^{\theta_0+\theta'}\right)}{e^{\theta_0} \big/ \left(1+e^{\theta_0}\right)} = e^{\theta'} \frac{1+e^{\theta_0}}{1+e^{\theta_0+\theta'}}.$$

Model ID	ϕ_1	ϕ_2	ϕ_3	ϕ_4	ϕ_5	ϕ_6	ϕ_7
$\mathcal{M}_0$	0	0	0	0	0	0	0
$\mathcal{M}_1$	15	15	15	0	0	0	0
$\mathcal{M}_2$	15	15	28	0	0	0	0
$\mathcal{M}_3$	15	15	15	6.6	0	0	0
$\mathcal{M}_4$	0	0	0	0	0	2.1	2.1
$\mathcal{M}_5$	0	0	0	0	0	2.1	4.2
$\mathcal{M}_6$	15	15	15	0	0	2.1	2.1

Table S2 Fold-increase values of ϕ_i (to two significant figures) which differentiate the different models, $\mathcal{M}_0, \cdots, \mathcal{M}_6$.

A. Simulation Framework

To simulate networks with nodel attributes and edges, we used the following approaches.

Simulating the adjacency matrix via a Bernoulli distribution

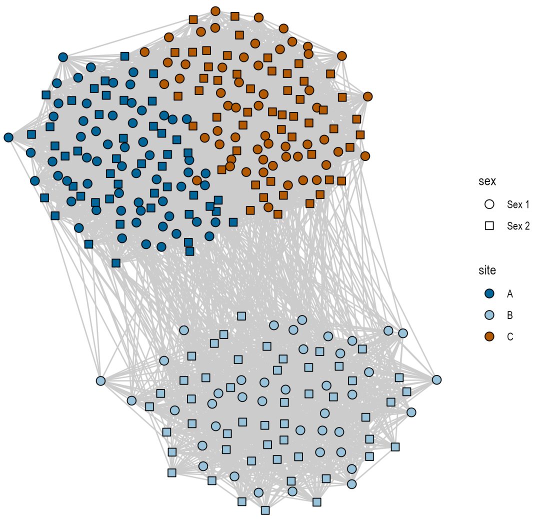

Figure S4 A single realisation of the site mix model, $\mathcal{M}_3$.

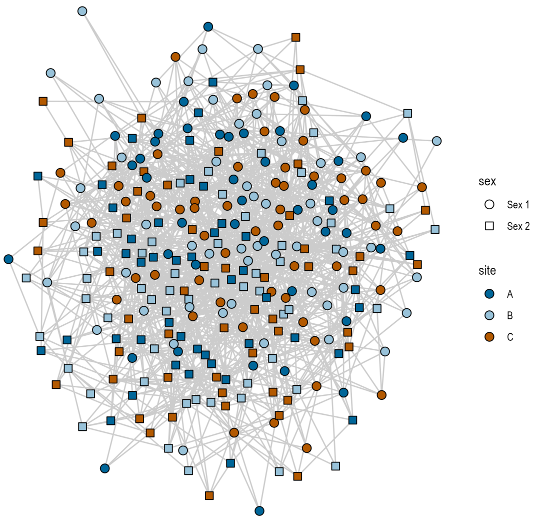

Figure S5 A single realisation of the sex match model, $\mathcal{M}_4$.

We begin by randomly sampling nodal attributes by assigning either “Site A”, “Site B” or “Site C” to each node with equal probability, and assigning either “Sex 1” or “Sex 2” with equal probability (or unequal probability when simulating unequal group sizes). Once each of the nodes has been randomly assigned both attributes, we could calculate the probability of an edge existing between nodes i and j, denoted p_{ij}, using the coefficient values in Table S1. We then sampled A_{ij} from a Bernoulli distribution of the form $A_{ij} \sim \text{Bern}(p_{ij})$, and set $A_{ij} = A_{ji}$.

When simulating missing edges, we simply added an additional final step where we randomly selected each existing edges to be removed with probability 0.05.

Simulating the network via a dyad-dependent Exponential Random Graph model

To simulate dyad-dependent networks, we used the *simulate.ergm()* function from the ergm R-package (Hunter *et al.* 2008), using the same model and coefficients as for the Bernoulli-based simulations. The clear difference is the need to supply a coefficient for the *triangles* term, denoted β_Δ, in the model. To estimate a realistic value for β_Δ, we considered an Approximate Bayesian Computational study of the empirical data. By simulating using the full model from the empirical data, we were able to look at how much dyad-dependence might be required in an example where the study was resolved to the pedigree-level (instead of a continental, inter-cultural network, for example). Hence, we believe that we are exploring the strongest effects of dyad-dependence that researchers are likely to experience in a similar study.

We took the number of nodes from the empirical network ($n = 202$), and applied the same site, sex/age, period and orientation observations to the nodes. Following this, we used the same coefficients estimated from the fitted dyad-independent ERGM (see Table 1), but simulated 10^4 possible values (blue shaded region S8) for the triangles coefficient such that it had prior distribution

$$\beta_\Delta \sim N(1,1). \tag{1}$$

We decided that β_Δ would likely be positive as an underlying pedigree is more likely to cause an increased likelihood of edge-dependence. We still allowed some prior density to be negative to show that these values were unrealistic.

We then used the *abc()* function from the abc R-package (Csilléry *et al.* 2012), comparing the observed to the simulated number of triangles, via the rejection method with a tolerance of 1%. The posterior distribution (red shaded region S8) deviates strongly from the prior, indicating a mean value for β_Δ of 0.3227, and a standard deviation of 0.0236. Importantly, both negative and “large” values of β_Δ were rejected. Finally, since the standard deviation was an order of magnitude lower than the mean, we decided to use the mean value of the posterior in the simulation study of the performance of our method and BIC to correctly identify the model of interest.

S4. Model selection comparison

To compare the performance of model selection methods on the same data we calculated the Akaike Information criterion (AIC), the Bayesian Information criterion (BIC) for every realisation. Additionally, we attempted to use the p-values of coefficients in the model summary as a model selection method, retaining models where coefficients had p-value less than 0.05, and hence selection this model (called p-value method from here on). For automated model selection we take the model with the smallest BIC, however when interpreting the BIC, it is important to consider the scale of the improvement in BIC for more complex models: a change in BIC of zero to two units is considered “not worth mentioning,” and hence models within two of each other are often considered equally valid (Drton and Plummer 2017).

BIC outperformed both AIC and the p-value method as it was 100% accurate for all 700 realisations. AIC performed reasonably, but less well, with an overall accuracy of 94.71%. However, using AIC led to the null model being misclassified as models with either site or sex as significant variables in 16% of simulations, which would lead to a high false positive rate when no variables are significant.

Conversely, the p-value method performed worse than either

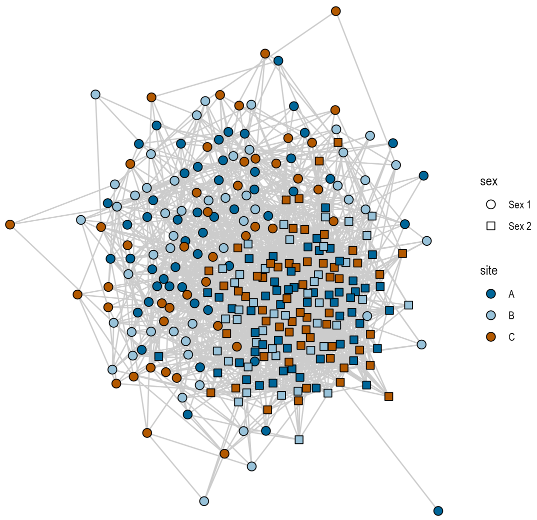

Figure S6 A single realisation of the differential sex match model, $\mathcal{M}_5$.

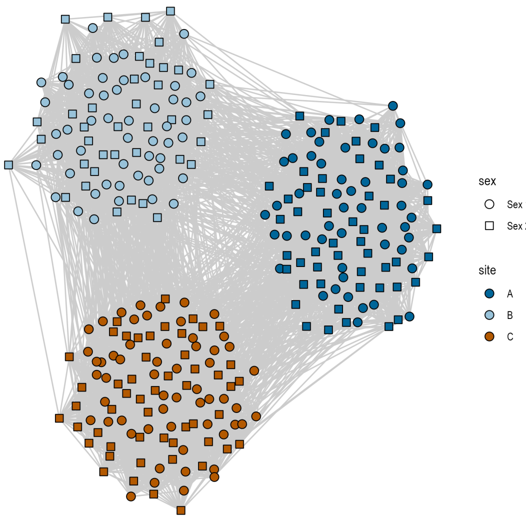

Figure S7 A single realisation of the mixed homophily model, $\mathcal{M}_6$.

information criterion approach. The p-value method achieved only 47% accuracy, and suffered from issues of false positive classification for the null model (as well as one-parameter models), as well as clearly being biased towards more complicated, higher-dimensional models when the correct variables were identified.

Hence, based on these results, we suggest the use of BIC for model selection when using ERGMs.

Finally, we simulated the number of models exactly as for the dyad-independent study except with the additional effect of dyad-dependency with $\beta_\Delta = 0.3227$ (see Section A). A confusion matrix of the results show (Figure S11) that, overall the method performs extremely well, again using BIC, even in the presence of dyad-dependence (estimated from empirical data). We note though that while the nature of the homophily was missclassified in the models where just site was significant (12% of differnetial nodematch and 2% of nodematch), that the correct variables were always returned.

A. Comparing values of AIC to AICc in our simulations

We also recorded the corrected AIC (AICc) for simulation, but found that the AIC and AICc were highly correlated ($r^2 = 1$), with an intercept of zero and a slope of one ($p < 2, 2 \times 10^{-16}$). This behaviour was not surprising as the AICc is a correction for the AIC to account for small sample sizes. The AICc is found via

$$\text{AICc} = \text{AIC} + \frac{2k^2 + 2k}{n - k - 1},$$

where n is the sample size, and k is the number of parameters in the model. Since the number of observed pairs, $n = \binom{N}{2}$, is large for networks with even relatively few nodes, N, the penalty term quickly tends to zero. Hence, we do not consider reporting the AICc as it is nearly identical to AIC (see Figure S12).

B. Model selection with addiitonal missing edges

To test the effect of missing edges due to stochastic noise, we performed a simulation study of model selection performance as before, but added that edges can also be “missed”, i.e., incorrectly infered to not exist, but removing edges with probability 0.05. We chose 0.05 as this is well within the expected probability for IBD inference when reasonable quality control thresholds are followed.

We followed the same methodology as for assessing model selection with BIC. We found that the confusion matrix was unchanged compared to Figure 1 in the main text, indicating that a false negative rate of approximately 5% does not appear to affect the performance of BIC model selection under the simulation scenarios tested in this study.

C. Calculating the expected number of edges in a network

Let a network have N nodes, and that node i has some known attribute, arbitrarily numbered $T_i \in \{1, \cdots, P\}$. Let the set S_k be the set of nodes with attribute k, i.e., $S_k = \{i | T_i = k\}$, where $|S_k| = m_k$. When comparing nodes, we can thus calculate that the number of pairwise comparisons between two sets of nodes with attribute types $T_i, T_j = 1, \cdots, P$, and $T_i \leq T_j$, will be

$$n_{T_iT_j} = \begin{cases} \binom{m_{T_i}}{2}, & T_i = T_j, \\ m_{T_i} m_{T_j}, & T_i \neq T_j. \end{cases} \quad (2)$$

Next, assume that the model for the log-odds of a connection between nodes with attributes $T_i, T_j \in \{1, \cdots, P\}$, for $T_i \leq T_j$, follows the equation

$$\log\left(\frac{p_{ij}}{1 - p_{ij}}\right) = \theta_0 + \theta_{T_iT_j},$$

meaning that

$$p_{ij} = \frac{1}{1 + e^{-(\theta_0 + \theta_{T_iT_j})}}.$$

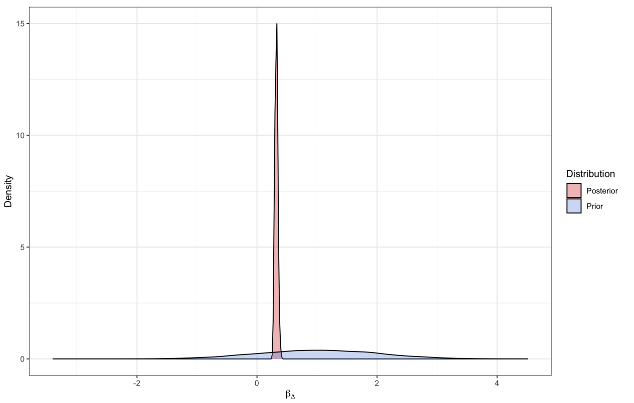

Figure S8 Approximate Bayesian computational approach to estimating the coefficient for triangles (β_Δ, x axis) in the dyad-depdendent simulation study. Fill colour indicates the prior distribution (blue) and the posterior distribution (red).

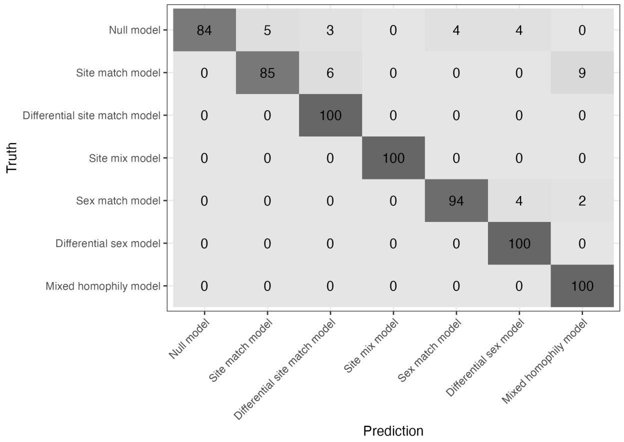

Figure S9 Confusion matrix for model selection accuracy using the Akaike Information criterion.

Finally, assume that we wish to know the number of edges present in the network, denoted A. These edges can be spearated into the edges between each attribute type, denoted $A_{T_i T_j}$, where $T_i \leq T_j$. Hence,

$$A = \sum_{\ell=1}^{P} \sum_{p=1}^{\ell} A_{T_\ell T_p}.$$

Hence, for E_{ij}, the random variables representing if an edge exists between nodes i and j, the expected number of edges in

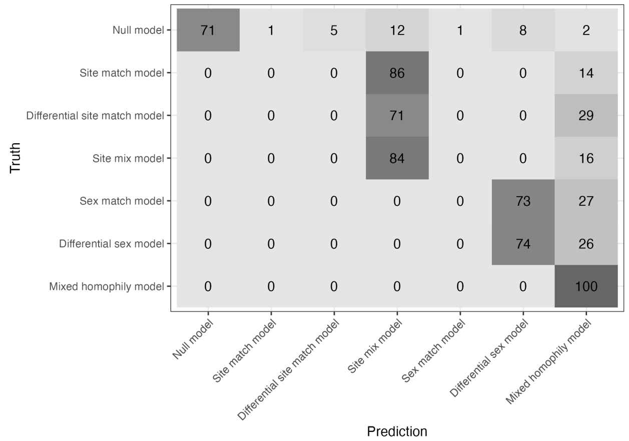

Figure S10 Confusion matrix for model selection accuracy using the p-value method.

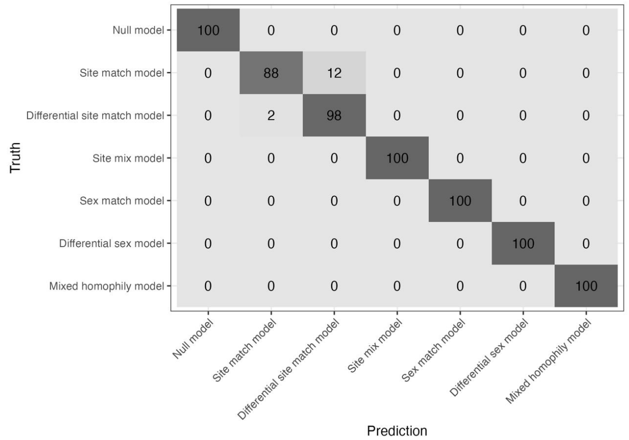

Figure S11 A confusion matrix for the accuracy of model selection via BIC with the presence of dyad-dependence.

the network is

$$\begin{aligned}
\mathrm{E}\left[A\right] &= \mathrm{E}\left[\sum_{\ell=1}^{P} \sum_{p=1}^{\ell} A_{T_\ell T_p}\right] \\
&= \sum_{\ell=1}^{P} \sum_{p=1}^{\ell} \mathrm{E}\left[A_{T_\ell T_p}\right] \\
&= \sum_{\ell=1}^{P} \sum_{p=1}^{\ell} \mathrm{E}\left[\frac{1}{2} \sum_{v_1 \in S_i} \sum_{v_2 \in S_j} \delta_{v_1 \neq v_2} E_{v_1 v_2}\right] \\
&= \sum_{\ell=1}^{P} \sum_{p=1}^{\ell} \frac{n_{\ell p}}{1 + e^{-(\theta_0 + \theta_{\ell p})}}
\end{aligned}$$

S5. Empirical data

A. Data description

The empirical data in this study comes from three (exhaustively-sampled) Avar-associated sites in Hungary: Hajdúnánás-Fürj-halom-járás (HNJ, $n = 11$, 567-700 AD), Kunszállás-Fülöpjakab (KFJ, $n = 37$, 601-700 AD), Kunpeszér-Felsőpeszéri út (KUP, $n = 13$, 601-700 AD) and Rákóczifalva Bagi-földek 8 (RK,

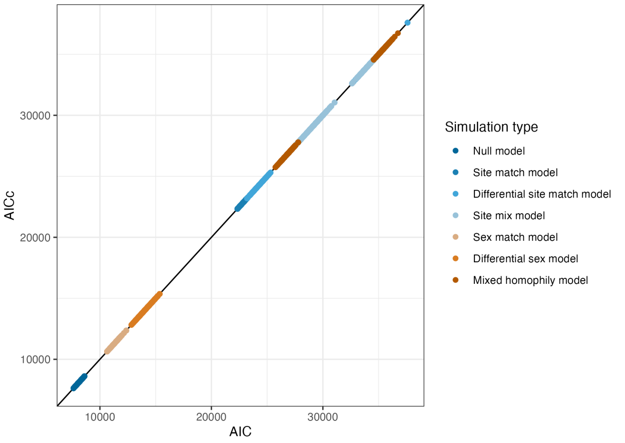

Figure S12 A linear regression of the corrected AIC (AICc) and the AIC, with colour indicating the simulation model, showing that AICc and AIC are perfectly correlated with an intercept of zero and a slope of one.

$n = 176$, 601-822 AD).The data was sequenced at the Max Planck Institute for Evolutionary Anthropology following standardized protocols designed in Ancient DNA Core facilityGnecchi-Ruscone *et al.* (2024). The sequence data was imputed using GLIMPSERubinacci *et al.* (2021) and IBD blocks were called using ancIBDRingbauer *et al.* (2023). For a full description of how the data was processed, see Gnecchi-Ruscone *et al.*Gnecchi-Ruscone *et al.* (2024).

We defined individuals as "related" if they shared at least two blocks of IBD of length 12cM, and at least one block of IBD of length 16cM, and who yielded a proportion of genotype posterior probabilities above the threshold of 0.99 for more than 70% of the imputed sites (frac_gp>0.7). The genetic sex of the individuals was estimated using the ratio of coverage on the X and Y chromosomes versus coverage on the autosomesGnecchi-Ruscone *et al.* (2024).

For each individual we recorded the site at which they were found ("HNJ", "KFJ", "KUP" or "RK"), the period in which they lived (Early Avar period "EA" or Middle/Late Avar period "MA/LA"), the combined genetic sex and age of the individual ("XX/Adult", "XX/Subadult", "XY/Adult" or "XY/Subadult"), the burial orientation of the individual ("N-S", "NNW-SSE" or "NW-SE"), whether the individual was buried with a seemingly random item in the form of an iron buckle ("Iron Buckle" or "No Iron Buckle"), and whether the individual was buried with an item that indicates high status in the form of horse riding equipment ("Belt/Harness" or "No Belt/Harness").

B. Sensitivity of ERGMs to changing the IBD cut off

In Section A we discuss that a cut off c is chosen to define when an edge exists in the network. This choice must be made by the researcher in the context of the research questions of interest. For example, if the IBD cut off is quite long, indicating that individuals must be *very* closely genetically related (say the first- or second-degree of relatedness), then this will produce a very different network to one where the cut off allows up to relatively distant degrees of relatedness, say at the limits of resolution of ancIBRingbauer *et al.* (2023) the seventh- to the tenth-degree. Neverthless, we explore how this changes the empirical analysis of the Avar netowrk presented in Section F by exploring cut offs based on T_8, the total sum of IBD blocks of at least 8cM in length.

We considered 20 log-uniformally spaced values of c between 100 and 2000. We chose these cut offs as the Ringbuaer *et al.* show via simulation that these values roughly equate to degrees-of-relatedness between the (distant) sixth- and (close) second-degrees, respectively. For each value of c we performed model selection with the same set of variables as we found in the full analysis (site, sex_age, orientation and period), selecting the model with the minimum BIC value. We also recorded the estimated coefficient in an ERGM using the final model selected in the empricial analysis in Section F of the main text.

When look at the results of the sensitivity analysis we notice that in all cases, no variable of interest was removed, albeit with minor changes in the complexity of the homophily/heterophily (Figure S13) The coefficient estimates for burial site visibly increase after the first four values of c, owing exclusively to the fact that after this value of c, there are no inter-site edges. Next we observe that for $c > 777$, that the best-fitting model prefers nodematch to nodemix for the period, as the intraconnectedness within periods settles to more similar levels for closer degrees of relatedness. Similarly, for $c > 1246$, the model selects nodediff over nodemix for age_sex. This reflects that the process of exogamy is likely more homogeneous at the closer degrees of relatedness, and loses the ability to detect deeper connections between adult female individuals who may share a deeper genetic relative in a breeding network.

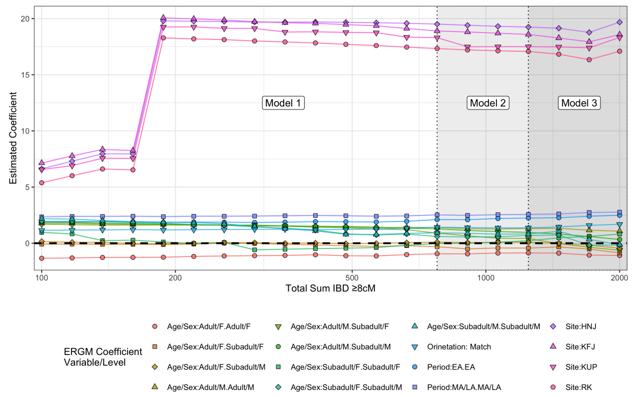

Figure S13 A sensitivity analysis of the coefficient estimates of the empirical data (y axis) to different choices of the cut off c (x axis) for defining an edge choice. The colours and shapes indicate different coefficients (and their levels) with the black dashed line indicating a value of zero. The different levels of background shading indicate when different models were prefered via BIC.
Model 1: nodemix('age_sex')+nodematch('period')+ nodematch('orientation')+nodematch('site',diff=TRUE)
Model 2: Model 1 + nodediff('period') → nodematch('period')
Model 3: Model 2 + nodemix('age_sex') → nodediff('age_sex')

C. Centrality measures

We began by clustering the vertices using the Louvain clustering algorithm (see Figure S14 A and B)Held *et al.* (2016). We observe that HNJ is separated into just two clusters, and that KUP and KFJ form two clusters each, with one being shared between them. Further, we see that RK forms nine clusters with three major clusters representing a large amount of within-site clustering that is not observed at the other sites.

We then inspected the connectivity in the model by calculating the degree centrality which measures the number of vertices each vertex is connected to (see Figure S14 C). We see that the main cluster at KFJ, and the three major sub-clusters at RK, indicate the highest amount of connectivity, indicating that these pedigrees and sites were simply larger.

Finally we looked at the betweenness centrality which measures vertices which are important to "connectedness" in the network (see Figure S14 D). A simple interpretation here would be high values of betweennes centrality indicate individuals who connect distinct, but connected, clusters. We find that the only obvious connecting individuals are within RK and KFJ, again connecting the four major clusters mentioned in the analysis of the degree centrality measures.

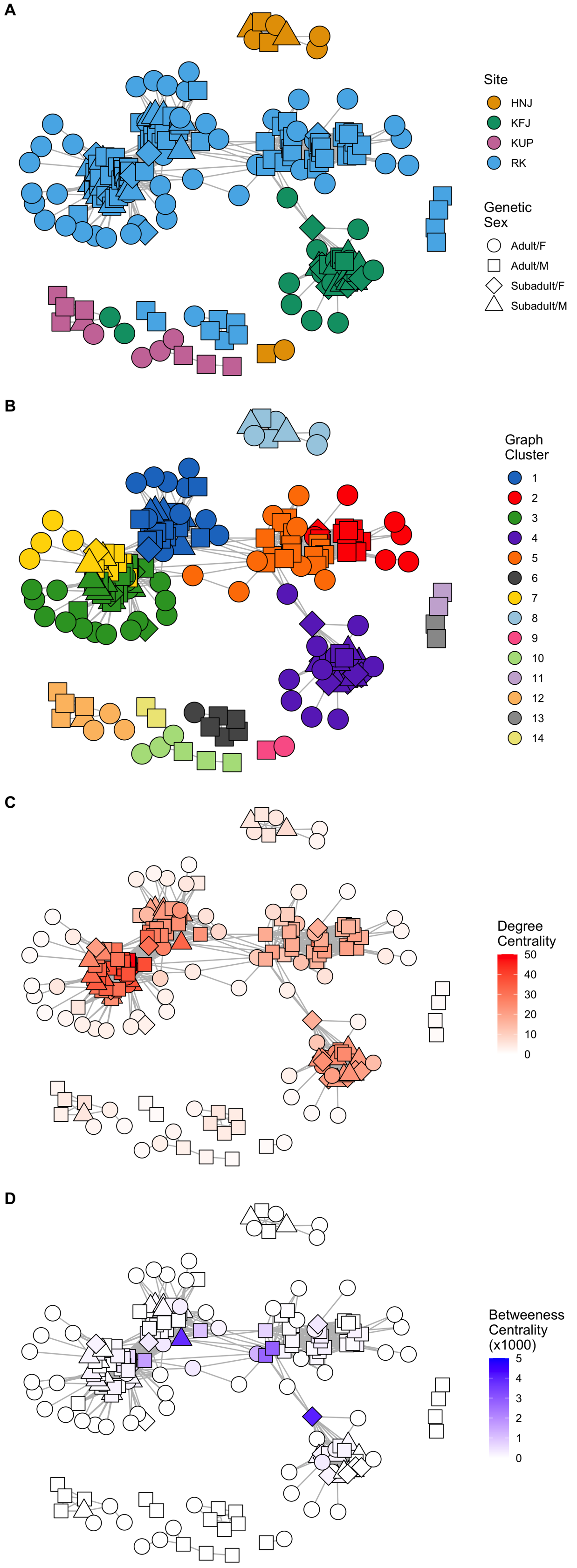

Figure S14 Network representations of the Avar data set with (A) site, (B) Louvain clustering, (C) degree centrality and (D) betweenness centrality indicated by vertex colours. Shape indicates the age and sex of the individuals.

Bibliography

Literature cited

Csilléry K, François O, Blum MG. 2012. abc: an R package for approximate Bayesian computation (ABC). Methods in ecology and evolution. 3:475–479.

Drton M, Plummer M. 2017. A Bayesian information criterion for singular models. Journal of the Royal Statistical Society Series B: Statistical Methodology. 79:323–380.

Gnecchi-Ruscone GA, Rácz Z, Samu L, Szeniczey T, Faragó N, Knipper C, Friedrich R, Zlámalová D, Traverso L, Liccardo S *et al.* 2024. Network of large pedigrees reveals social practices of Avar communities. Nature. 629:376–383.

Held P, Krause B, Kruse R. 2016. Dynamic clustering in social networks using louvain and infomap method. In: . pp. 61–68. IEEE.

Hunter DR, Handcock MS, Butts CT, Goodreau SM, Morris M. 2008. ergm: A package to fit, simulate and diagnose exponential-family models for networks. Journal of statistical software. 24:nihpa54860.

Ringbauer H, Huang Y, Akbari A, Mallick S, Patterson N, Reich D. 2023. ancIBD-screening for identity by descent segments in human ancient DNA. BioRxiv. .

Rubinacci S, Ribeiro DM, Hofmeister RJ, Delaneau O. 2021. Efficient phasing and imputation of low-coverage sequencing data using large reference panels. Nature genetics. 53:120–126.
